# RACE DIFFERENCES IN ALLOSTATIC LOAD AMONG BLACK AND WHITE MEN: DOES AGE MATTER?

**DOI:** 10.1093/geroni/igz038.2615

**Published:** 2019-11-08

**Authors:** Roland J Thorpe, Hossein Zare, Paul Archibald, Marino A Bruce, Keith Norris, Keith E Whitfield

**Affiliations:** 1 Johns Hopkins Bloomberg School of Public Health, Baltimore, Maryland, United States; 2 Sr. Programmer, JHU, Baltimore, Maryland, United States; 3 Morgan State University, Baltimore, Maryland, United States; 4 Vanderbilt University, Nashville, Tennessee, United States; 5 UCLA, Los Angeles, California, United States; 6 Wayne State University, Detroit, Michigan, United States

## Abstract

Although Black-White disparities in health and mortality among men persist, there is a paucity of work focusing on race differences in physiological dysregulation of biological processes resulting from the cumulative impact of stressors among men. The purpose of this study was to assess potential race differences in Allostatic Load (AL) among adult men and if such differences vary by age. Data were drawn from the 1999-2010 NHANES, and the study population included 2700 non-Hispanic Black (NHB) and 19930 Non-Hispanic White (NHW) born in US. AL was derived by summing across cardiovascular, metabolic, and inflammatory biomarkers considered to be high risk, resulting in a count variable ranging from 0 to 9. Race was based on self-report. Age was categorized: 18-24, 25-44, 45-64, and 65 years and older. Negative binomial regression was used to examine the relationship between race and AL score. Models included education, marital status, family income, health insurance and self-reported health. Adjusting for potential confounders, NHB men had a higher AL score ((incidence rate ratio (IRR) = 1.06, 95% confidence interval (CI) 1.01, 1.11) than NHW men. NHB men 25-44 years old had a higher AL score than (IRR = 1.14, 95% CI;1.04, 1.24) than their NHW peers. No race differences with respect to AL score were observed among the other age groups. Race differences in AL vary by age categories. Efforts to improve longevity should focus on developing age-tailored health promoting strategies to reduce stress among Black men during early adulthood.

